# Supplementary material for: Association between eating behavior and the immediate neural activity caused by viewing food images presented in and out of awareness: A magnetoencephalography study
Source: PLoS One. 2022 Dec 29;17(12):e0275959. doi: 10.1371/journal.pone.0275959 (PMC9799321; doi:10.1371/journal.pone.0275959)
Supplement: Attachment — Submitted filename: Ishida_Food_PLOSO_Response.docx [file pone.0275959.s001.docx]

**Responses to the comments**

**Reviewer 1**

1. The statement on lines 54-58 is an intriguing possibility but only one reference was given in support of it and that reference does not provide any support for the notion that unconscious perception of food stimuli helps explain the poor outcomes of behavioral weight loss programs. The authors’ hypotheses about unconscious reactions to food need to be recognized as highly speculative at this point and not try to oversell (here and elsewhere in the manuscript) that these effects are even real, never mind relevant to weight control. This literature is still very much in the “proof of principle” stage and the authors’ narrative throughout their paper should reflect this.
**Response:**

**Thank you for the suggestion. We added the descriptions in the Discussion to clearly state that the idea that appetitive behavior is driven more by unconscious decision-making processes than by the conscious decision-making processes is speculative at this point: “the idea that appetitive behavior is driven more by unconscious decision-making processes than by the conscious ones is speculative at this point and our present study is in the proof of principle stage only to show the potential importance of unconscious neural processes in eating behavior” (lines 486 to 489, in the revised manuscript).**

2. A major issue throughout the manuscript is that the authors do not realize that food has both specific and non-specific effects on physiology. As a highly rewarding substance, food creates general arousal (and, for dieters, may create an approach-avoidance conflict) above and beyond its specific appetitive effects. So in their discussion of the literature and also in their study, they should discuss that fact that general arousal could account for some or all of the effects they are currently attributing to food. Ideally the authors would have utilized a second control condition, using another type of arousing stimulus like sexually arousing pictures. This limitation needs to be recognized throughout the paper.
**Response:**

**We agree with the Reviewer 1 that** **we are not able to exclude the possibility that the effects caused by viewing the food images might have been due to arousal induced by the images in our present study; however, we think that it is of value to assess the neural effects caused by food images including those related to arousal induced by the images when we assess the neural activity caused by viewing food images because arousal can inevitably be induced by viewing food and/or food images in our daily lives. We added the descriptions, “It is of note that the effects caused by viewing the food images may include those of arousal induced by the images (i.e.,** **another arousing stimulus other than food images was not employed as a control stimulus in our present study)” (lines 103 to 105, in the revised manuscript) and “Third, since another arousing stimulus other than food images was not employed as a control stimulus in our present study, we are not able to exclude the possibility that the effects caused by viewing the food images might have been due to arousal induced by the images in our present study; however, we think that it is valuable to assess the neural effects caused by food images including those related to arousal induced by the images when we assess the neural activity caused by viewing food images because arousal can inevitably be induced by viewing food and/or food images in our daily lives” (lines 465 to 472, in the revised manuscript).**

3. In several places in the paper, the authors make statements about their own or others’ research findings without specifying whether the results were based on unconscious or conscious stimuli (e.g., lines 90-93). Please revise accordingly.
**Response:**

**Thank you for the suggestion. We revised our manuscript as suggested by the Reviewer 1 (lines 90 to 91 and lines 92 to 93, in the revised manuscript).**

4. The authors often refer to the regulation of eating or the measurement of food intake, but they are simply referring to scores on self-report measures like emotional eating. The validity of these questionnaires is questionable and so the authors should instead be referring, for example, to “scores on measures of emotional eating,” not to “eating behavior” or “eating regulation.”
**Response:**

**We revised our manuscript as suggested by the Reviewer 1 (lines 83, 84, 354, 356, 359, 381 to 382, 437, 440, 442, 444, 446, and 448, in the revised manuscript).**

5. The authors need to explain why they didn’t present visible and invisible stimuli in the same session (e.g., lines 117-119).
**Response:**

**We wanted to confirm that our participants were not aware of the food and object images in the invisible trials and were aware of the food and object images in the visible trials by asking them whether they recognized images other than the mask image afterward and therefore, if the visible and invisible stimuli were randomly presented in the same session, we had to ask our participants whether they recognized images other than the mask image after each trial, resulting in much longer experimental time period for each participant, which is not optimum for MEG data collection. We added the description, “Since we wanted to confirm that our participants were not aware of the food and object images in the invisible trials and were aware of the food and object images in the visible trials by asking them whether they recognized images other than the mask image afterward, as described below, the visible and invisible stimuli were not presented in the same session” (lines 122 to 126, in the revised manuscript).**

6. Lines 144-146 and elsewhere: what were the “mask pictures” of?
**Response:**

**The mask picture was the same for every trial across the food and object sessions in the visible and invisible conditions: The mask picture was created by arranging colored lines and circles and did not created by processing specific picture. We added the sentence, “The mask picture was the same for every trial across the food and object sessions in the visible and invisible conditions: The mask picture was created by arranging colored lines and circles and did not created by processing specific picture” (lines 155 to 157, in the revised manuscript), in the Materials and methods.**

7. No criteria were given for why certain Japanese foods were used as stimuli. How were they chosen? Were they known to represent highly palatable foods?
**Response:**

**The set of food pictures used in our present study was identical to those used in our previous studies (Yoshikawa et al., 2013, 2014a, 2014b; Takada et al., 2018; Nakamura et al., 2020). We chose food items which are thought to be consumed in daily living in Japan, including highly palatable food such as steak, hamburger, pizza, fried rice, and takoyaki (octopus balls). We revised the description in the Materials and methods (lines 158 to 161, in the revised manuscript)**

**References:**

**1. Yoshikawa T, Tanaka M, Ishii A, Watanabe Y. Immediate neural responses of appetitive motives and its relationship with hedonic appetite and body weight as revealed by magnetoencephalography. Medical science monitor : international medical journal of experimental and clinical research. 2013;19:631-40. Epub 2013/08/03. doi: 10.12659/MSM.889234. PubMed PMID: 23907366; PubMed Central PMCID: PMC3737122.**

**2. Yoshikawa T, Tanaka M, Ishii A, Watanabe Y. Suppressive responses by visual food cues in postprandial activities of insular cortex as revealed by magnetoencephalography. Brain Res. 2014;1568:31-41. Epub 2014/04/29. doi: 10.1016/j.brainres.2014.04.021. PubMed PMID: 24768717.**

**3. Yoshikawa T, Tanaka M, Ishii A, Fujimoto S, Watanabe Y. Neural regulatory mechanism of desire for food: revealed by magnetoencephalography. Brain Res. 2014;1543:120-7. Epub 2013/11/13. doi: 10.1016/j.brainres.2013.11.005. PubMed PMID: 24216133.**

**4. Takada K, Ishii A, Matsuo T, Nakamura C, Uji M, Yoshikawa T. Neural activity induced by visual food stimuli presented out of awareness: a preliminary magnetoencephalography study. Scientific reports. 2018;8(1):3119. Epub 2018/02/17. doi: 10.1038/s41598-018-21383-0. PubMed PMID: 29449657; PubMed Central PMCID: PMC5814400.**

**5. Nakamura C, Ishii A, Matsuo T, Ishida R, Yamaguchi T, Takada K, et al. Neural effects of acute stress on appetite: A magnetoencephalography study. PLoS One. 2020;15(1):e0228039. Epub 2020/01/23. doi: 10.1371/journal.pone.0228039. PubMed PMID: 31968008; PubMed Central PMCID: PMCPMC6975544.**

8. A major problem with the study is that it included a measure of heart rate variability and of brain electrical activity but there was little explanation of why these two physiological measures were chosen or how they might relate to one another. They chose a measure of heart rate variability and, though there is a large literature on the relation between HRV and appetitive stimuli, almost none of this was included. Further, please explain what is thought to reflect healthy or unhealthy heart rate variability and why this might be related to perception of food stimuli.
**Response:**

**In our previous study (Takada et al., 2018), we hypothesized that if food pictures presented below the threshold of awareness play important roles such that affecting appetitive control and/or eating behavior, food pictures presented below the threshold of awareness may cause alterations of autonomic activity. In fact, it has been reported that, in a study in which affective pictures presented both below and above the threshold of awareness, alteration in heart rate variability was induced by both presentation conditions (Bulut et al., 2018). As a result, we showed that the LF/HF ratio was increased by viewing food pictures presented below the threshold of awareness in our previous study (Takada et al., 2018) and confirmed this finding in our present study, suggesting that the food pictures can affect neural activity even if presented below the threshold of awareness. As the Reviewer 1 suggested, there have been reports on the relation between HRV and appetitive stimuli. For example, it has been reported that food stimuli presented above the threshold of awareness caused increased low frequency (LF) power of heart rate variability in healthy participants (Nederkoorn et al., 2000) and that individuals with obesity showed altered vagal reactivity to food stimuli presented above the threshold of awareness compared with that of healthy individuals (Udo et al., 2014 and Spitoni et al., 2017). Therefore, there may be a possibility that the alterations in heart rate variability caused by food pictures presented below the threshold of awareness are related to the disorders related to appetitive control including obesity; however, further studies are needed on this point. We added the descriptions, “We hypothesized that if food pictures presented below the threshold of awareness play important roles such that affecting appetitive control and/or eating behavior, food pictures presented below the threshold of awareness may cause alterations of autonomic activity. In fact, it has been reported that, in a study in which affective pictures presented both below and above the threshold of awareness, alteration in heart rate variability was induced by both presentation conditions (Bulut et al., 2018). As a result, we showed that the LF/HF ratio was increased by viewing food pictures presented below the threshold of awareness in our previous study (Takada et al., 2018) and confirmed this finding in our present study, suggesting that the food pictures can affect neural activity even if presented below the threshold of awareness. Since it has been reported that food stimuli presented above the threshold of awareness caused increased low frequency (LF) power of heart rate variability in healthy participants (Nederkoorn et al., 2000) and that individuals with obesity showed altered vagal reactivity to food stimuli presented above the threshold of awareness compared with that of healthy individuals (Udo et al., 2014 and Spitoni et al., 2017), there may be a possibility that the alterations in heart rate variability caused by food pictures presented below the threshold of awareness are related to the disorders related to appetitive control including obesity; however, further studies are needed on this point”, in the Discussion (lines 404 to 419, in the revised manuscript).**

**References:**

**1. Takada K, Ishii A, Matsuo T, Nakamura C, Uji M, Yoshikawa T. Neural activity induced by visual food stimuli presented out of awareness: a preliminary magnetoencephalography study. Scientific reports. 2018;8(1):3119. Epub 2018/02/17. doi: 10.1038/s41598-018-21383-0. PubMed PMID: 29449657; PubMed Central PMCID: PMC5814400.**

**2. Bulut NS, Wurz A, Yorguner Kupeli N, Carkaxhiu Bulut G, Sungur MZ. Heart rate variability response to affective pictures processed in and outside of conscious awareness: Three consecutive studies on emotional regulation. Int J Psychophysiol. 2018;129:18-30. Epub 2018/05/23. doi: 10.1016/j.ijpsycho.2018.05.006. PubMed PMID: 29787784.**

**3. Nederkoorn C, Smulders FT, Jansen A. Cephalic phase responses, craving and food intake in normal subjects. Appetite. 2000;35(1):45-55. Epub 2000/07/18. doi: 10.1006/appe.2000.0328. PubMed PMID: 10896760.**

**4. Udo T, Weinberger AH, Grilo CM, Brownell KD, DiLeone RJ, Lampert R, et al. Heightened vagal activity during high-calorie food presentation in obese compared with non-obese individuals--results of a pilot study. Obes Res Clin Pract. 2014;8(3):e201-98. Epub 2014/05/23. doi: 10.1016/j.orcp.2013.05.006. PubMed PMID: 24847667; PubMed Central PMCID: PMCPMC4031442.**

**5. Spitoni GF, Ottaviani C, Petta AM, Zingaretti P, Aragona M, Sarnicola A, et al. Obesity is associated with lack of inhibitory control and impaired heart rate variability reactivity and recovery in response to food stimuli. Int J Psychophysiol. 2017;116:77-84. Epub 2017/04/10. doi: 10.1016/j.ijpsycho.2017.04.001. PubMed PMID: 28390903.**

9. On lines 263-267, the authors should analyze these data with repeated measures ANOVAs, not separate t-tests.
**Response:**

**According to the Reviewer 1’s suggestion, we added the results of repeated measures ANOVA. We added the description, “Two-way repeated-measures analysis of variance (ANOVA) was performed to assess alterations in the LF/HF ratio caused by viewing the food pictures compared with viewing the object pictures in the visible and invisible conditions” (lines 268 to 271, in the revised manuscript), in the Materials and methods and revised the descriptions in the Results as follows: “****Two-way repeated-measures ANOVA was performed to assess alterations in the LF/HF ratio caused by viewing the food pictures compared with viewing the object pictures in the visible and invisible conditions. A main effect of pictures (i.e., food or object pictures) was identified [F(1, 11) = 21.637, *P* = 0.001]. There was no main effect of conditions (i.e., visible or invisible conditions) [F(1, 11) = 0.431, *P* = 0.525] and no interaction was apparent [F(1, 11) = 0.984, *P* = 0.343]” (lines 286 to 291, in the revised manuscript).**

10. I have never heard of analyzing the “intensity of an interaction.” The reason an interaction is interesting is because it captures different responses on a dependent variable depending on the level of an independent variable. One would presumably want to separately capture both patterns that comprise an interaction. What does intensity of an interaction even mean and is there precedent in the literature for using it? Also, I have no idea what the relationships in Figure 4 refer to because the “strength of the interaction” has no apparent meaning.
**Response:**

**Thank you for the suggestion. The descriptions regarding this point were inadequate in our previous manuscript: We showed the existence of the brain regions which responded differently to the food pictures between the visible and invisible conditions. As shown in Figure 3B and 3C, the neural activities caused by viewing the food and object pictures were sharply different between the visible and invisible conditions (please refer to the Response to the Reviewer 1’s comment #14). Since the extent to which the neural responses to the pictures in the visible condition was different from those in the invisible condition was variable between participants, we wanted to assess whether the extent to which the neural responses to the pictures in the visible condition was different from those in the invisible condition was related to the indices of eating behavior. We assessed the extent to which the neural responses to the pictures in the visible condition was different from those in the invisible condition by the values calculated as the contrast of the levels of oscillatory brain activity [(food pictures in visible condition + object pictures in invisible condition) – (object pictures in visible condition + food pictures in invisible condition)] for each participant, corresponding to the interactions assessed in the SPM analyses. We added the description, “The extent to which the neural responses to the pictures in the visible condition was different from those in the invisible condition was assessed by the values calculated as the contrast of the levels of oscillatory brain activity [(food pictures in visible condition + object pictures in invisible condition) – (object pictures in visible condition + food pictures in invisible condition)] for each participant, corresponding to the interactions assessed in the SPM analyses”, in the Materials and methods (lines 233 to 238, in the revised manuscript). We revised the expressions “the intensity of the interaction” and “the interaction” to be “the extent to which the neural responses to the pictures in the visible condition was different from those in the invisible condition” in our revised manuscript (lines 352 to 359, lines 364 to 370, lines 379 to 380, lines 435 to 437, lines 437 to 438, lines 446 to 447, and Figure 4).**

11. There was no mention of counterbalancing stimuli. Also, if conscious food stimuli were presented first, couldn’t that suggest to participants what the invisible stimuli were?
**Response:**

**As described in the Materials and methods (lines 126 to 127, in the revised manuscript) and shown in Figure 1, our experiment was performed in a four-crossover fashion (i.e., approximately half of the participants performed the visible condition before performing the invisible condition). We analyzed data from the participants who declared that they had not recognized even the existence of any pictures other than the mask picture in the invisible condition and the participants were not informed of the existence of pictures other than the mask picture in the invisible condition before the stat of the experiment. Therefore, all the participants whose data were analyzed seemed to have believed that the mask image and the fixation cross were the only images presented during the invisible condition.**

12. The fact that a large fraction of the participants claimed to have seen the “unconscious” food stimuli and therefore had to be eliminated is a major methodological flaw that the authors do not seem to recognize. This suggests that they did not pilot test their subliminal presentation of stimuli to see if they “worked.” Those who did and did not recognize the food stimuli should be compared on all measures to see if they differed in any way (using effect sizes rather than significance levels, since the sample sizes are so small).
**Response:**

**We intentionally employed longer duration for the presentation of the food and object pictures than our previous study (Takada et al., 2018), which led to the increased number of the participants who recognized the food and/or object pictures in the invisible condition, because we wanted to ensure that, in the visible condition, the food and object pictures presented for the same duration to that in the invisible condition could be recognized by most of the participants. In addition, since we planned to perform group analyses of the MEG data, we also wanted to use the same duration for the presentation of the food and object pictures for every participant. Therefore, the MEG data for the participants who recognized no food or object pictures under the invisible condition (and recognized all the food and object pictures under the visible condition) were analyzed in our present study.**

**According to the suggestion made by the Reviewer 1, we compared the participants whose MEG data were analyzed with those whose MEG data were not analyzed on the measures obtained in our present study (i.e., the indices related to eating behavior; Table 1). We added the descriptions, “The indices related to eating behavior assessed by using TFEQ (i.e., uncontrolled eating, cognitive restraint, and emotional eating) in the participants whose MEG data were analyzed and those whose MEG data were not analyzed were shown (Table 1)” and “The effect sizes comparing the participants whose MEG data were analyzed with those whose MEG data were not analyzed on the indices related to eating behavior were calculated by using G*Power software (Version 3. 1. 9. 2; Faul et al., 2007)”, in the Results (lines 313 to 316, in the revised manuscript) and Materials and methods (lines 278 to 281, in the revised manuscript), respectively. We also added the sentences in the Discussion as follows: “Since we wanted to ensure that, in the visible condition, the food and object pictures presented for the same duration to that in the invisible condition could be recognized by most of the participants and thus the duration for the presentation of the food and object pictures was longer than that would have been able to make the pictures invisible for most of the participants in the invisible condition, only the MEG data for the participants who recognized no food or object pictures under the invisible condition (and recognized all the food and object pictures under the visible condition) were analyzed. Although the relationship between the individual differences on whether the masked food pictures are recognized or not in the invisible condition and those of the indices of eating behavior is unknown, our results (Table 1) suggest the possibility that the differences in the indices of eating behavior have some influence on the visibility of the masked food pictures. Further study is needed on this point” (lines 472 to 482, in the revised manuscript).**

**References:**

**1. Takada K, Ishii A, Matsuo T, Nakamura C, Uji M, Yoshikawa T. Neural activity induced by visual food stimuli presented out of awareness: a preliminary magnetoencephalography study. Scientific reports. 2018;8(1):3119. Epub 2018/02/17. doi: 10.1038/s41598-018-21383-0. PubMed PMID: 29449657; PubMed Central PMCID: PMC5814400.**

**2. Faul F, Erdfelder E, Lang AG, Buchner A. G*Power 3: a flexible statistical power analysis program for the social, behavioral, and biomedical sciences. Behavior research methods. 2007;39(2):175-91. Epub 2007/08/19. PubMed PMID: 17695343.**

13. The authors discuss findings in high-gamma range at length but do not mention why no findings existed for low- or medium-level gamma. What might this signify?
**Response:**

**As the Reviewer 1 suggested, the findings were observed only in high-gamma band frequency. On the one hand, this suggests the importance of high-gamma band neural activity in investigating the conscious and unconscious neural processes related to eating behavior. On the other hand, we think it is premature to state the differential roles of neural activities in eating behavior between high-gamma and low- and medium-gamma frequency bands. We added the description, “The interactions were observed only in high-gamma band frequency. This suggests the importance of high-gamma band neural activity in investigating the conscious and unconscious neural processes related to eating behavior; however, further studies were needed to completely understand the differential roles of neural activities in eating behavior between high-gamma band and gamma bands 1 and 2”, in the Discussion (lines 449 to 453, in the revised manuscript).**

14. I do not understand the potential meaning of the sharply different patterns of gamma in Figures 3 A and B. It seems to me that what we have here are findings that transcend any appetitive model or theory of why this pattern may exist. In other words, just because our technical capabilities can detect differential brain responding doesn’t mean that the field has sufficient knowledge to interpret what these responses might mean in terms of cognition or behavior. Unless the authors have a better speculation about the meaning of their findings than what they currently provide, this may be the best conclusion they can reach at present.

**Response:**

**We hypothesized that there may be an interaction between the conscious and unconscious neural process related to eating behavior if the unconscious neural process related to eating behavior has effects which are not explained by the conscious one and we showed the existence of the brain regions which responded differently to the food pictures between the visible and invisible conditions in our present study (Figure 3A and Table 2). The neural activity caused by viewing each visual stimulus (i.e., the food and object pictures in the visible and invisible conditions) in the each brain region which responded differently to the food pictures between the visible and invisible conditions was shown in Figure 3B and 3C: The neural activities caused by viewing the pictures were sharply different between the visible and invisible conditions as the Reviewer 1 suggested. Since the extents of the differences in the neural activity caused by viewing the pictures between the visible and invisible conditions were related to the indices of eating behavior, it can be thought that the differences in the neural activity caused by viewing the pictures seem to have some influences on eating behavior. However, as the Reviewer 1 suggested, we were not able to refer to the precise mechanisms by which the differences in the neural activities shown in Figure 3B and 3C affect eating behavior at this time. We revised the descriptions in the Discussion as follows: “we could not determine the causal association between the differences of the neural responses to the pictures between the visible and invisible conditions and the indices of eating behavior and the precise mechanisms by which the differences in the neural activities caused by viewing the food pictures between the visible and invisible conditions affect eating behavior if the differences in the neural activities affect eating behavior in this study” (lines 441 to 446, in the revised manuscript).**

**END**
