# Supplementary material for: Association between eating behavior and the immediate neural activity caused by viewing food images presented in and out of awareness: A magnetoencephalography study
Source: PLoS One. 2022 Dec 29;17(12):e0275959. doi: 10.1371/journal.pone.0275959 (PMC9799321; doi:10.1371/journal.pone.0275959)
Supplement: Attachment — Submitted filename: Ishida_Responses_Revise2_CLT.docx [file pone.0275959.s002.docx]

**Responses to the comments**

**Reviewer** 2

1. In Line 261, it is described that LF and HF power were measured in absolute units (ms^2^). Using MEG system, the units seem to V^2^. Please confirm.

**Response:**

**The unit for the R-R intervals is millisecond (ms) and thus, the unit for the power spectral density of the R-R wave intervals is ms^2^/Hz. Therefore, the unit for LF and HF power is ms^2^, as described in the manuscript (line 280, in the revised manuscript).**

2. In Table 1, it is described, “Values are shown as mean ± SD”. Period is required.

**Response:**

**Thank you for the suggestion. We revised the point suggested by the Reviewer 2 (line 340, in the revised manuscript).**

3. In Line 344, it is described, “Only significant change is shown…” This may mean, “Only the results of the brain locations in which statistically significant changes were shown…”

**Response:**

**Thank you for the suggestion. We revised the sentence to be “Only the results of the brain locations in which statistically significant changes were observed were shown” (lines 368 to 369, in the revised manuscript).**

**Reviewer 3**

If the manuscript is revised, I recommend the authors include the 13 Ss who were dropped for potentially detecting (at least one) stimulus during invisible conditions. Awareness can be included as an ordinal factor based on the number of detections across invisible conditions. Then, if authors observe a negative association between BA activations and increasing awareness, their case for 'unconscious' processes would be strengthened.

**Response:**

**Thank you for the suggestion. However, since our present study was not designed to determine the number of the food and/or object items each participant might have recognized in the invisible condition (i.e., They answered whether they recognized at least one image other than the mask picture after each session and therefore, the frequency they might have recognized the items was not assessed. Likewise, we could not determine whether the items they might have recognized were the food pictures or object pictures: Some participants seemed to have exclusively recognized the object pictures and some participants seemed to have exclusively recognized the food pictures, and some participants seemed to have recognized both the food and object pictures), we are not able to include the number of detections across the invisible conditions as a variable in our analysis assessing the interaction effect.**

Introduction:

1) l. 58 - Discussions of how unconscious processes may influence eating behaviors, outlined in Amd and Baillet (2019) and Passarelli et al (2022), imply food-related stimuli can elicit unconscious affective and motivational responses without being coupled to propositional knowledge. This provides a potential mechanism as to how appetitive behaviors can be unconsciously moderated, which could be discussed here.

**Response:**

**Thank you for the useful comment. We added the descriptions in the Introduction as follows: “In fact, it has been demonstrated that the greater saliva production and the increased rating of hunger were caused by coupling the food-related stimuli presented below the threshold of awareness with positively valenced terms, suggesting that the unconscious processes, which would not evoke eating-related deliberation, can effectively modulate affective and motivational responses caused by food-related stimuli (Amd et al., 2019 and Passarelli et al., 2022)” (lines 58 to 62, in the revised manuscript).**

**References:**

1. **Amd M, Baillet S. Neurophysiological Effects Associated With Subliminal Conditioning of Appetite Motivations. Frontiers in psychology. 2019;10:457. Epub 2019/03/21. doi: 10.3389/fpsyg.2019.00457. PubMed PMID: 30890986; PubMed Central PMCID: PMCPMC6411685.**
2. **Passarelli DA, Amd M, de Oliveira MA, de Rose JC. Augmenting salivation, but not evaluations, through subliminal conditioning of eating-related words. Behav Processes. 2022;194:104541. Epub 2021/11/24. doi: 10.1016/j.beproc.2021.104541. PubMed PMID: 34813914.**

2) l. 70 – How was the "threshold of awareness" assessed?

**Response:**

**In their study (Takada et al., 2018), it was confirmed that the participants whose data were analyzed did not recognized any images other than the mask-pictures by a questionnaire. We revised the descriptions, “Compared with a condition in which mosaic pictures created from original food stimuli were presented below the threshold of awareness, the presentation of the food stimuli below the threshold of awareness caused the activation of sympathetic nervous activity and alterations in neural activity in Brodmann’s areas (BA) 47 and 13, which are related to the alteration of sympathetic nervous activity and the level of cognitive restraint of food intake, respectively”, in the Introduction of the previous manuscript to be “Compared with a condition in which mosaic pictures created from original food stimuli were presented so as not to be recognized by the participants, the food stimuli presented so as not to be recognized by them caused the activation of sympathetic nervous activity and alterations in neural activity in Brodmann’s areas (BA) 47 and 13, which are related to the alteration of sympathetic nervous activity and the level of cognitive restraint of food intake, respectively” (lines 71 to 76, in the revised manuscript).**

**References:**

1. **Takada K, Ishii A, Matsuo T, Nakamura C, Uji M, Yoshikawa T. Neural activity induced by visual food stimuli presented out of awareness: a preliminary magnetoencephalography study. Scientific reports. 2018;8(1):3119. Epub 2018/02/17. doi: 10.1038/s41598-018-21383-0. PubMed PMID: 29449657; PubMed Central PMCID: PMC5814400.**

3) l. 95 – Expand on why this onset window is notable – e.g., activations before 200-300 ms are likely to be pre-lexical, meaning they should be minimally reflective of top-down/propositional/conscious control (Amd & Baillet, 2019; Bayer et al., 2019)

**Response:**

**Thank you for the suggestion. We added the descriptions, “In addition, it has been reported that lexical processing and thus the activation of meaning seem to start after around 200 ms of stimulus onset (Barber et al., 2007; Amd et al., 2019; Bayer et al., 2019), suggesting that the unconscious processing of the presented stimulus is performed within the time window around 0-200 ms from stimulus onset (lines 99 to 102, in the revised manuscript).**

**References:**

1. **Barber HA, Kutas M. Interplay between computational models and cognitive electrophysiology in visual word recognition. Brain Res Rev. 2007;53(1):98-123. Epub 2006/08/15. doi: 10.1016/j.brainresrev.2006.07.002. PubMed PMID: 16905196.**
2. **Amd M, Baillet S. Neurophysiological Effects Associated With Subliminal Conditioning of Appetite Motivations. Frontiers in psychology. 2019;10:457. Epub 2019/03/21. doi: 10.3389/fpsyg.2019.00457. PubMed PMID: 30890986; PubMed Central PMCID: PMCPMC6411685.**
3. **Bayer M, Grass A, Schacht A. Associated valence impacts early visual processing of letter strings: Evidence from ERPs in a cross-modal learning paradigm. Cognitive, affective & behavioral neuroscience. 2019;19(1):98-108. Epub 2018/10/21. doi: 10.3758/s13415-018-00647-2. PubMed PMID: 30341624.**

Materials & Methods:

4) l. 153 – Both the visible and invisible conditions presented food stimuli for 33.4 ms, with the latter condition associated with a backward mask in the location of the food stimuli. Because stimulus location was held constant, how do we know response effects were not influenced by the moving mask instead of unconscious appetite-related processes? A better control would have been to increase SOAs for the visible condition or present fixation/stimulus near the bottom half of the screen followed by a mask that moves towards the bottom end of the screen. This should be described under limitations.

**Response:**

**Since we thought it was suboptimal to increase the duration of the presentation of the food or object images in the visible condition in that the duration of the presentation of the food or object images in the visible condition was not the same as that in the invisible condition, which might have caused the difference in the neural responses between the conditions, we decided to use the visual presentation of which the time course of the presentation was the same as that in the invisible condition in the visible condition as described in our manuscript. However, this procedure (i.e., the procedure used in our present study) was also suboptimal in that the vertical position of the mask image in the visible condition was not the same as that in the invisible condition as the Reviewer 3 suggested; however, since we assessed the alterations in neural responses corresponding to [(food pictures in visible condition + object pictures in invisible condition) – (object pictures in visible condition + food pictures in invisible condition)] in our present study, the effect caused by the mask image was canceled out in the analysis (i.e., the contrast described above was equal to [(food pictures in visible condition – object pictures in visible condition) – (food pictures in invisible condition – object pictures in invisible condition]). We mentioned this point in the Discussion: “the vertical position of the mask image in the visible condition was not the same as that in the invisible condition because we wanted the time course of the presentation in the visible condition to be the same as that in the invisible condition; however, since we assessed the alterations in neural responses corresponding to [(food pictures in visible condition + object pictures in invisible condition) – (object pictures in visible condition + food pictures in invisible condition)] in our present study, the effect caused by the mask image was canceled out in the analysis (i.e., the contrast described above was equal to [(food pictures in visible condition – object pictures in visible condition) – (food pictures in invisible condition – object pictures in invisible condition]) and therefore, the effect caused by the difference of the position of the mask picture between the conditions seems to be minimal in our present study” (lines 530 to 540, in the revised manuscript).**

**As for the suggestion made by the Reviewer 3 that “present fixation/stimulus near the bottom half of the screen followed by a mask that moves towards the bottom end of the screen” may be an alternative approach for preparing the control condition. Thank you for the suggestion.**

5) l. 161 – Was caloric quantity included as a covariate? Lee et al (2022) showcased high-caloric foods engage attentional processes significantly faster than low-caloric foods

**Response:**

**Thank you for the suggestion. The caloric quantity was not included as a covariate in our present study: For the MEG data analyses performed in our present study, it is necessary to increases the frequency of the presentation of each food item (i.e., each food item should be presented more than 50 times, considering the exclusion of the trials due to the contamination with noises) in order to assess the neural activity caused by each food item to include the caloric quantity as a covariate. We would like to assess the effect caused by the caloric quantity of each food item in the future study designed to assess the neural activity caused by each food item.**

6) l. 162 – Were the same items repeated across invisible and visible conditions? How would you account for memory effects for participants exposed to a visible->invisible sequence?

**Response:**

**The items presented in the invisible condition were the same as that presented in the visible condition; however, since the contrast corresponding to [(food pictures in visible condition – object pictures in visible condition) – (food pictures in invisible condition – object pictures in invisible condition] was analyzed in our present study as described in the manuscript (lines 530 to 540, in the revised manuscript), the memory effect such that pointed out by the Reviewer 3 were expected to be canceled out in the process of the calculation of the contrast (i.e., the possible effects caused by the visible > invisible and/or the invisible > visible sequences existed both in the food and object sessions). We added the descriptions as follows: “Likewise, although the items presented in the invisible condition were the same as that presented in the visible condition, since the contrast corresponding to [(food pictures in visible condition – object pictures in visible condition) – (food pictures in invisible condition – object pictures in invisible condition] was analyzed in our present study, the memory effect were expected to be canceled out in the process of the calculation of the contrast (i.e., the possible effects caused by the visible > invisible and/or the invisible > visible sequences existed both in the food and object sessions)” (lines 541 to 547, in the revised manuscript).**

7) l. 167 – How was the picture "identical" to the mask? Why wasn't the actual mask used? Why were the stimulus and the (pseudo?) mask simultaneously presented (instead of a forward/backward sandwich procedure?)

**Response:**

**The picture co-presented with the food and object pictures was completely the same as the mask picture (i.e., the actual mask was used). The co-presented mask picture was not an indispensable component of the visual stimuli in that the co-presented mask picture was presented both in the visible and invisible conditions and therefore, the effect caused by the co-presented picture might have been canceled out between the conditions. The reason why we added this co-presented mask picture was to attenuate the potential effect caused by the difference of the number of the recognized images between the visible and invisible conditions: On the one hand, the fixation cross, the food or object items, and the mask picture were recognized in the visible condition and the fixation and the mask picture were recognized in the invisible condition if the mask picture was not co-presented. On the other hand, the fixation cross, the food or object items, and the mask picture were recognized in the visible condition and the fixation, co-presented mask picture, and the mask picture were recognized in the invisible condition if the mask picture was co-presented. We added the sentences, “The picture co-presented with the food and object pictures was completely the same as the mask picture (i.e., the actual mask was used). The reason why we added this co-presented mask picture was to attenuate the potential effect caused by the difference of the number of the recognized images between the visible and invisible conditions: On the one hand, the fixation cross, the food or object items, and the mask picture were recognized in the visible condition and the fixation and the mask picture were recognized in the invisible condition if the mask picture was not co-presented with the food or object pictures. On the other hand, the fixation cross, the food or object items, and the mask picture were recognized in the visible condition and the fixation, co-presented mask picture, and the mask picture were recognized in the invisible condition if the mask picture was co-presented with the food or object pictures”, in the Materials and methods (lines 177 to 187, in the revised manuscript).**

8) l. 174 – Fixation/stimulus always appeared near the top half of the screen?

**Response:**

**As the Reviewer 3 suggested, the fixation and the food or object items were always presented near the bottom of the upper half of the screen. We added the sentence, “The fixation and the food or object items were always presented near the bottom of the upper half of the screen”, in the Materials and methods (lines 191 to 192, in the revised manuscript).**

9) l. 279 – Who were the Ss' whose MEG data were not analyzed and why?

**Response:**

**We added the descriptions in the Materials and methods as follows: “As described in the Results, the MEG data from 17 participants were excluded from the analysis because of the contamination of the MEG data by the magnetic noise originating from outside the shielded room, the insufficient backward masking effect, and so on” (lines 299 to 301, in the revised manuscript).**

Results:

10) l. 289 – No effect of the visibility manipulation then

**Response:**

**One of the aims of our present study was to examine whether visual food stimuli affect the central nervous system even if presented below the threshold of awareness. Although, as the Reviewer 3 suggested, the effect of visibility was not observed in our present study, we were able to show that the visual food stimuli affected the central nervous system even if presented below the threshold of awareness in our present study.**

11) l. 295 – Include panel labels for visible and invisible conditions

**Response:**

**Thank you for the suggestion. We added the labels for visible and invisible conditions in Figure 2.**

12) l. 302 – Had the 13 participants who were dropped for stimulus recognition exposed to a visible>invisible sequence? A 40% attrition rate is quite high, and counts against the 'invisibility' of the food stimuli. Additionally, the awareness questionnaire was provided at the end of the task, but this may not capture conscious appraisal that was short-lived after stimulus onsets. A trial-by-trial awareness check could be more reliable and would probably increase attrition (Newell & Shanks, 2014)

**Response:**

**Twelve participants of the 13 participants pointed out by the Reviewer 3 were dropped for stimulus recognition in the invisible condition (i.e., One participant of the 13 participants was dropped because of the failure in recognizing at least one food or object items in the visible condition): Among the 12 participants, eight participants performed a visible > invisible sequence and four participants performed an invisible > visible sequence. We added the description, “Among the 12 participants who were dropped for stimulus recognition in the invisible condition, eight participants performed a visible > invisible sequence and four participants performed a invisible > visible sequence”, in the Results (lines 326 to 329, in the revised manuscript). For the purpose of reference, the null hypothesis that there was no relationship between the sequence of the conditions (i.e., a visible > invisible or an invisible > visible) and the visibility was not rejected (χ^2^ test, *P* = 0.183).**

**If the participants were asked whether they recognized images other than the mask picture after each trial, the experimental time period for each participant would have been much longer than that in our present study (i.e., since the number of the trials in each session was 300 in our present study, the experimental time period for each session would be 15 min longer than our present study if participants answered whether they recognized the images other than mask picture after each trial in 3 s):** **Since longer experimental time period is not optimum for MEG data collection** **from the perspective of minimizing the body movement during the data collection and the burden of participants,** **we decided to ask them whether they recognized images other than the mask picture afterward.** **To minimize the possibility that the MEG data of the participants who in fact recognized some of the food or object items in the invisible condition were included in our analysis,** **we excluded the MEG data of the participants who declared that they might have seen images other than the mask picture at least one time in the invisible condition regardless of whether they were able to mention what they saw or whether the items they mentioned were actually presented in the session. We added the description on this point as follows: “In addition, since longer experimental time period is not optimum for MEG data collection** **from the perspective of minimizing the body movement during the data collection and the burden of participants,** **we decided to ask them whether they recognized images other than the mask picture after each session.** **To minimize the possibility that the MEG data of the participants who in fact recognized some of the food or object items in the invisible condition were included in our analysis,** **we excluded the MEG data of the participants who declared that they might have seen items other than the mask picture at least one time in the invisible condition regardless of whether they were able to mention what they saw or whether the items they mentioned were actually presented in the session” (lines 522 to 530, in the revised manuscript).**

13) l. 314 – I do not understand the point of Table 1. Are the effect sizes representative of the mean differences between participant groups whose data were analyzed vs not analyzed? I struggle to infer the relevance of (say) participants' whose data were not analyzed having higher 'emotional eating' scores relative to participants whose data were analyzed

**Response:**

**The effect sizes are the representative of the mean differences between participant groups whose data were analyzed vs. not analyzed. As the Reviewer 3 pointed out, although this table may suggest the possibility that individuals with high emotional eating scores have tendency of recognizing masked food pictures easier than those with low emotional eating score as described in the Discussion (lines 517 to 522, in the revised manuscript), Table 1 seems not to be directly relevant to the interpretation of our present results. If the Reviewer 3 suggest that Table 1 should be deleted from our manuscript, we would like to delete Table 1 and related discussions.**

14) l. 357 – Change the x-axis labels of Figure 4 to be succinct. E.g., Panel A x-label could be 'BA-45 Z-scores'. The responses for emotional eating should be standardized before regression.

**Response:**

**Thank you for the suggestion. We revised the x-axis labels of Figure 4 as suggested by the Reviewer 3. We used the standardized values for emotional eating and cognitive restraint in the revised Figure. We added the description, “The standardized values for the indices of emotional eating and cognitive restraint were used”, in the legend of Figure 4 (lines 390 to 391, in the revised manuscript).**

Discussion:

15) l. 369 – you cannot make claims of 'awareness thresholds' given the limitations noted above

**Response:**

**Thank you for the suggestion. We revised the sentences to be, “In this study, participants viewed food and object pictures under visible and invisible conditions, respectively, and the neural activities over viewing the pictures were assessed by MEG”, in the Discussion (lines 393 to 395, in the revised manuscript).**

16) l. 381 – You cannot claim "could not have been" – participants may have been aware of the stimuli at the time of presentation but this had extinguished by the time of assessment.

**Response:**

**We revised the second paragraph of the Discussion as follows: “The data from individuals who answered that there was at least one picture in which they could not recognize a food item or object under the visible condition, and those from individuals who answered that there was at least one picture in which they recognized a food item or an object under the invisible condition were excluded from our analyses. Therefore, the participants whose data were analyzed reported that they have recognized food and object items under the visible condition and have not recognize food and object items under the invisible condition” (lines 404 to 410, in the revised manuscript).**

17) l. 397 – why would sympathetic activity not be viewed during visible conditions then?

**Response:**

**Thank you for the suggestion. The sentence in the previous manuscript pointed out by the Reviewer 3 was misleading. Therefore, we revised the sentence as follows: “By using the pictures of objects (i.e., not the mosaic pictures) as control stimuli in our present study, we could confirm that the increase of the sympathetic nervous activity caused by viewing the food pictures presented using the backward masking procedure reported in the previous study was not due to the difference between objects and non-objects” (lines 419 to 423, in the revised manuscript). The condition in which the food and mosaic pictures were presented without the backward masking procedure was not employed in the previous study. We mentioned the alteration of the sympathetic nervous activity caused by viewing the food pictures in the visible conditions observed in our present study in the Discussion (lines 440 to 448, in the revised manuscript).**

18) l. 409, 411 – Both groups showed variance in response to food stimuli above awareness thresholds. It is unclear why would there would "be a possibility" that HRV would similarly vary in the presence of food stimuli presented below awareness. One possibility is that 'partial' awareness of the food stimuli may be sufficient to generate full-scale representations (in which case observed effects would not really be reflective of 'unconscious processes' – cf. Kouider et al., 2010).

**Response:**

**Thank you for the suggestion. We added the descriptions, “There was no difference in the alteration of the sympathetic nervous activity caused by viewing the food pictures between the invisible and visible conditions (i.e., the main effect of conditions was not identified) in our present study, suggesting that the sympathetic nervous activity was increased by viewing the food pictures both in the visible and invisible conditions; however, since the participants whose data were analyzed reported that they did not recognize the food and object pictures exclusively in the invisible condition and the neural response to the visual food stimuli in the invisible condition was different from that in the visible condition as described below, it is speculated that the neural mechanisms by which the alteration of the sympathetic nervous activity was caused by viewing the food pictures in the invisible condition were not the same as those in the visible condition”, in the Discussion (lines 440 to 449, in the revised manuscript).
The partial awareness hypothesis (Kouider et al., 2010) suggested by the Reviewer 3 predicts that one feature may reach consciousness while others do not and it is postulated that individuals have high confidence about the informational content at some restricted levels in the state of partial awareness. Although we are not able to exclude the possibility that the visual food stimuli of which individuals reported not to be recognized worked as the sufficient stimuli to induce the alteration of the sympathetic nervous activity in the invisible condition, since there was no evidence that some of the features of the food stimuli presented in the invisible condition reached consciousness of our participants whose data were analyzed, it may be that the alteration of the sympathetic nervous activity caused in the visible condition was related to the unconscious processing of the food stimuli in the visible condition rather than that the alteration of the sympathetic nervous activity caused in the invisible condition was due to the existence of partial awareness of the food stimuli in the invisible condition, if this is the case. We added the descriptions, “Although we are not able to exclude the possibility that the visual food stimuli of which individuals reported not to be recognized worked as the sufficient stimuli to induce the alteration of the sympathetic nervous activity in the invisible condition, since there was no evidence that some of the features of the food stimuli presented in the invisible condition reached consciousness of our participants whose data were analyzed, it may be that the alteration of the sympathetic nervous activity caused in the visible condition was related to the unconscious processing of the food stimuli in the visible condition rather than that the alteration of the sympathetic nervous activity caused in the invisible condition was due to the existence of partial awareness of the food stimuli in the invisible condition (Kouider et al., 2010), if this is the case”, in the Discussion (lines 449 to 458, in the revised manuscript).**

**Reference:**

1. **Kouider S, de Gardelle V, Sackur J, Dupoux E. How rich is consciousness? The partial awareness hypothesis. Trends Cogn Sci. 2010;14(7):301-7. Epub 2010/07/08. doi: 10.1016/j.tics.2010.04.006. PubMed PMID: 20605514.**

19) l. 439-448 – It is not clear what you're trying to say. The sentence from l. 442-448 is particularly unwieldy

**Response:**

**Thank you for the suggestion. We revised the descriptions pointed out by the Reviewer 3 as follows: “These findings indicate that it is essential to taking both the neural responses to visual food stimuli presented above the threshold of awareness and those below the threshold of awareness into consideration to understand the neural mechanisms related to eating behavior, although we could not determine the causal association between the existence of the interaction in the oscillatory brain activity and the specific features of eating behavior (i.e., emotional eating and cognitive restraint) in our present study**

**” (lines 481 to 487, in the revised manuscript).**

20) l. 481 – how would indices of eating behavior influence food visibility?

**Response:**

**Thank you for the suggestion. We revised the sentence pointed out by the Reviewer 3 as follows: “Our result (Table 1) may suggest that individuals with high emotional eating scores have tendency of recognizing masked food pictures easier than those with low emotional eating score; however, since this was not confirmed in our present study and the neural mechanisms by which individual differences in eating behavior affect the visibility of the masked food pictures are unknown, further study is needed on this point” (lines 517 to 522, in the revised manuscript).**

21) l. 487 –See the discussion in Passarelli et al 2022 to extend this speculation

**Response:**

**Thank you for the suggestion. We revised the descriptions regarding the speculation pointed out by the Reviewer 3 as follows: “Although our present study is in the proof of principle stage only to show the potential importance of unconscious neural processes in eating behavior, together with the reports that unconscious decision-making processes have effects on appetitive behavior mentioned in the Introduction (Amd et al., 2019 and Passarelli et al., 2022), our findings can be expected to motivate further studies seeking to clarify the neural mechanisms related to eating behavior from the perspectives of conscious and unconscious neural processes” (lines 551 to 556, in the revised manuscript). Please refer to the response to the Reviewer 3’s comment #1.**

**References:**

1. **Amd M, Baillet S. Neurophysiological Effects Associated With Subliminal Conditioning of Appetite Motivations. Frontiers in psychology. 2019;10:457. Epub 2019/03/21. doi: 10.3389/fpsyg.2019.00457. PubMed PMID: 30890986; PubMed Central PMCID: PMCPMC6411685.**
2. **Passarelli DA, Amd M, de Oliveira MA, de Rose JC. Augmenting salivation, but not evaluations, through subliminal conditioning of eating-related words. Behav Processes. 2022;194:104541. Epub 2021/11/24. doi: 10.1016/j.beproc.2021.104541. PubMed PMID: 34813914.**

**END**
