# Supplementary material for: Association between eating behavior and the immediate neural activity caused by viewing food images presented in and out of awareness: A magnetoencephalography study
Source: PLoS One. 2022 Dec 29;17(12):e0275959. doi: 10.1371/journal.pone.0275959 (PMC9799321; doi:10.1371/journal.pone.0275959)
Supplement: Attachment — Submitted filename: Ishida_Revise_3.doc [file pone.0275959.s003.doc]

September 2, 2022

The Editor, *PLOS ONE*

Dear Editor:

Thank you so much for your letter concerning our manuscript, entitled "Association between eating behavior and the immediate neural activity caused by viewing food images presented in and out of awareness: a magnetoencephalography study" (PONE-D-20-30494R2). After carefully reading your letter and the comments of the reviewers, we have made the revisions for the manuscript. Our point-by-point responses to the comments are provided on separate pages. My responses following the reviewers’ comments are bolded. New words added to the manuscript are indicated in the quotations and presented in green color in the manuscript. We would like to thank the reviewers for their suggestions and the editors for arranging and reviewing our manuscript. We think that the manuscript has been significantly improved.

In addition, we would like to update our financial disclosure as follows:

"AI: JSPS KAKENHI Grant No. 16H03248 and TY: JSPS KAKENHI Grant No. 22K11732"

Thank you so much again for your constructive comments.

Sincerely yours,

Akira Ishii, M.D., Ph.D.

Department of Sports medicine,

Osaka City University Graduate School of Medicine

1-4-3 Asahimachi, Abeno-ku, Osaka 545-8585, Japan

Tel: +81-6-6645-3711

Fax: +81-6-6645-3712

E-mail: a.ishii@med.osaka-cu.ac.jp

**Responses to the comments**

**Reviewer 2**

We have been requested to respond to the comment of Reviewer #2; however, the comment of Reviewer #2 seems not to require additional responses (i.e., Reviewer #2 mentioned “This paper is well written and the authors seems to adequately respond to the Reviewer s’ comments, I think that this paper is acceptable for the publication of PLOS ONE”). Please let us know if there still be the comments to be responded.

**Reviewer 3**

Thank you for the opportunity to review the revised manuscript. The revisions have made the arguments and data presentation clearer, for which I commend the authors. However, a number of earlier issues remain a concern, and prevent me from recommending acceptance of the current manuscript. If the manuscript is revised, I recommend the participants dropped for recognizing images be included as a control.
**Response:**

**Thank you for reviewing our manuscript. As we have mentioned in the previous response to the Reviewer 3’s comment #12, we intended to minimize the possibility that the MEG data of the participants who in fact recognized some of the food or object items in the invisible condition were included in our analysis, we excluded the MEG data of the participants who declared that they might have seen images other than the mask picture at least one time in the invisible condition regardless of whether they were able to mention what they saw or whether the items they mentioned were actually presented in the session. In other words, to exclude for certain the MEG data of the participants who recognized at least one food or object item in the invisible condition, we accepted the possibility that the MEG data of the participants who did actually recognized no items in the invisible condition were excluded (i.e., the group of the participants who were dropped for recognizing images seems to include both those who recognized some of the food and/or object items in the invisible condition and those who did not actually. It is of note that the participants who were dropped for recognizing images did not necessary recognize every item in the invisible condition, if they recognized some of the items in the invisible condition). Therefore, since the comparison between the MEG data of the participants who were included in our analyses and those of the participants who were excluded from our analyses would not properly reflect the difference of the neural activity between the conscious and unconscious recognition, it is not appropriate to compare the MEG data of the participants who were included in our analyses with those of the participants who were excluded from our analyses.**

**In addition, the between-individual comparison that contrasts the MEG data of the participants who were included in our analyses with those of the participants who were excluded from our analyses, suggested by the Reviewer 3, may reflect the neural activity related to the individual difference in the susceptibility to the backward masking. Of course, the neural basis of the susceptibility to the backward masking is an interesting topic: however, since the aim of our present study was not focused on the susceptibility to the backward masking, we think it is not appropriate to include the participants who were dropped for recognizing images as a control in our present study, even if the participants whose MEG data were excluded certainly recognized every item in the invisible condition.**

1. The exclusion of 12 participants remains a serious concern. I understand you cannot discriminate which items participants viewed, but you could still include the 12 as a control. Replicating (or not) your outcomes with the dropped sample will inform the reader of the manipulation check's validity. e.g. assuming you replicate the analyses shown in Fig 2 across the control, the awareness checks would be validated and discussions re absence of awareness vindicated. Alternatively, overlapping MEG activations between your active and control groups would suggest awareness checks were not capturing the desired construct adequately. Either finding would bolster the Results and Discussion. Dropping the participants from analysis prevents (dis)confirming those claims.
**Response:**

**Thank you for the suggestion. As we have described above, we think it is not appropriate to include the participants who were dropped for recognizing images in our present study as a control (i.e., Since our experiment was not designed to analyze the MEG data of the participants who were dropped for recognizing images, the data from excluded participants were not comparable to those from included participants). Instead of including the factors related to the visibility of each item into the analyses as the Reviewer 3 have suggested, we carefully designed our experiment to improve the quality and reliability of our experimental data as much as possible: We concentrated on minimizing the possibility that the MEG data of the participants who recognized any of the food or object items in the invisible condition were included in our analysis and therefore, the exclusion of 12 participants was not out of the scope of our assumption.**

2. Given the limitations pointed out across lines 518-522, the effect sizes reported in Table 1 are still not readily interpretable - perhaps high emotional eating corresponds with greater image sensitivity overall, regardless of image content? The MEG outcomes of the control group would allow for parallel contrasts (e.g., (dis)similar activation between Invisible/Active and Control participants) and better inform the relevance of the effects.
**Response:**

**Thank you for the suggestion. We think it is not appropriate to include the participants who were dropped for recognizing images in our present study as a control as we have described above. In addition, we agree with the Reviewer 3 in that Table 1 in the previous manuscript was not readily interpretable and we think that Table 1 seems not to be directly related to the interpretation of our present results, we decided to delete Table 1 and the related descriptions in the revised manuscript.**

3-1. Writing need to be concise. Many statements meander e.g., across lines 50-54:
"While education on diet and/or behavioral treatments for obesity are important approaches to control body weight and prevent the health problems caused by obesity [10-12],several studies have reported that regardless of these approaches, diet adherence remains low [13-15], and nearly half of patients undergoing behavioral treatment for obesity return to their original weight within 5 years of the end of the treatment [16]. "
This could be condensed to:
"Educational and/or behavioral interventions for addressing obesity are partially successful, with nearly half of treated patients returning to pre-treatment weights within 5 years of intervention completion [13-16]."
**Response:**
**Thank you for the suggestion. We revised the sentence in the Introduction: “Educational and/or behavioral interventions for addressing obesity are partially successful, with nearly half of treated patients returning to pre-treatment weights within 5 years of intervention completion (Harris et al., 1996; Burke er al., 1997; Nelson et al., 2002; Butryn et al., 2011)**” **(lines 50 to 52, in the revised manuscript).**

**References:**

**1. Harris MI. Medical care for patients with diabetes. Epidemiologic aspects. Annals of internal medicine. 1996;124(1 Pt 2):117-22. Epub 1996/01/01. doi: 10.7326/0003-4819-124-1_part_2-199601011-00007. PubMed PMID: 8554202.**

**2. Burke LE, Dunbar-Jacob JM, Hill MN. Compliance with cardiovascular disease prevention strategies: a review of the research. Ann Behav Med. 1997;19(3):239-63. Epub 1997/07/01. doi: 10.1007/BF02892289. PubMed PMID: 9603699.**

**3. Nelson KM, Reiber G, Boyko EJ, Nhanes, III. Diet and exercise among adults with type 2 diabetes: findings from the third national health and nutrition examination survey (NHANES III). Diabetes Care. 2002;25(10):1722-8. Epub 2002/09/28. doi: 10.2337/diacare.25.10.1722. PubMed PMID: 12351468.**

**4. Butryn ML, Webb V, Wadden TA. Behavioral treatment of obesity. Psychiatr Clin North Am. 2011;34(4):841-59. Epub 2011/11/22. doi: 10.1016/j.psc.2011.08.006. PubMed PMID: 22098808; PubMed Central PMCID: PMCPMC3233993.**

3-2. Similarly, the following passage is difficult to follow:
"On the one hand, the fixation cross, the food or object items, and the mask picture were recognized in the visible condition and the fixation and the mask picture were recognized in the invisible condition if the mask picture was not co-presented with the food or object pictures. On the other hand, the fixation cross, the food or object items, and the mask picture were recognized in the visible condition and the fixation, co-presented mask picture, and the mask picture were recognized in the invisible condition if the mask picture was co-presented with the food or object pictures."
Do you mean to say something like:
"Fixation and mask stimuli appeared at supraliminal (>34 ms) visual thresholds across visible and invisible conditions. Food/object targets appeared at supraliminal thresholds during visible conditions, and at subliminal (<34 ms) thresholds during invisible conditions"?
**Response:**
**We revised the sentences in the Materials and Methods as follows: “The participants would recognize the fixation, the food or object items, and the mask pictures in the visible condition and they would recognize the fixation and the mask picture in the invisible condition if the mask picture was not co-presented with the food or object pictures (i.e., the number of the images recognized by the participants would be three in the visible condition and that would be two in the invisible condition)” (lines 178 to 183, in the revised manuscript).**

4. - Head movement is a concern during MEG, and I agree that 300 trials are too much for trial-by-trial verbal reports. This could be addressed in a future work by either i) including a check at random intervals (although this will leave possibility of detection during unassessed trials...) or ii) incorporating a forced-choice awareness check after some/all trials, which would not require head movement.

**Response:**

**Thank you for the useful comment. We will consider the suggested approaches in our future studies.**

**Reviewer 4**

1. 170 - Food and object pictures were same in visible and invisible conditions. Even though the mean interval between two visits was approximately 1 week, participants might memorize the pictures after the visible condition visit. Since 12 participants recognized the food object pictures under the invisible condition, why not use different food and object pictures for visible and invisible conditions?
**Response:**

**Thank you for the comment. While using the same pictures both in the visible and invisible conditions is beneficial for adjusting the characteristics of the visual stimuli across the conditions this may be the cause of the recognition of the food and object pictures in the invisible condition if the participants memorized** **the pictures after the visible condition visit, as the Reviewer 4 suggested.**

**Among the 12 participants who were dropped for stimulus recognition, eight participants performed a visible > invisible sequence and four participants performed an invisible > visible sequence; however, since the null hypothesis that there was no relationship between the sequence of the conditions (i.e., a visible > invisible or an invisible > visible) and the visibility was not rejected (χ^2^ test, *P* = 0.183), the memory effect seems not to have been the primary cause of the recognition of the food and/or object pictures in the invisible condition. In addition, as we have mentioned in the Discussion (lines 525 to 530, in the revised manuscript), since the contrast corresponding to [(food pictures in visible condition – object pictures in visible condition) – (food pictures in invisible condition – object pictures in invisible condition] was analyzed in our present study, the memory effect was expected to be canceled out in the process of the calculation of the contrast.**

2. 329 - Was there any motion effects in the MEG data collection?

**Response:**
**As we described in the Results, the MEG data from four participants were excluded due to the contamination with magnetic noise originating from outside the shielded room. We are not able to exclude the possibility that the noise caused by the motion effects were included in the magnetic noise; however, any noise which seemed to be related to bodily motion (and that seemed to be caused by other reasons) were not observed in the MEG data included in our analysis.**

3. 353 - In figure 3 (B) and (C), was there any difference between the BA 45 and 47 in high-gamma oscillatory brain activity?

**Response:**

**Since two peaks, which were in the BA 45 and 47, were detected in the cluster in the left frontal region shown in Figure 3A in the SPM analysis and the Z-values in these peaks were similar to each other (i.e., 4.19 and 4.18, respectively), we reported the levels of increases in high-gamma oscillatory brain activity both in the BA 45 and 47 in Figure 3B and 3C, respectively. We think there are no substantial differences in the level of alterations in high-gamma oscillatory brain activity between the BA 45 and 46 (Figure 3B and 3C, and Table 1, in the revised manuscript).**

**END**
